# Effect of Gabapentin on Heart Rate Variability in Patients with Painful Diabetic Peripheral Neuropathy: A Double-blinded Randomized Controlled Trial

**DOI:** 10.5812/ijpr-165080

**Published:** 2025-10-10

**Authors:** Farnaz Ahmadi, Maryam Vasheghani, Mehdi Moradi, Jalal Poorolajal, Fatemeh Zeraati, Hamidreza Dezfouli, Shadi Shafaghi

**Affiliations:** 1 Lung Transplantation Research Center, National Research Institute of Tuberculosis and Lung Diseases (NRITLD), Shahid Beheshti University of Medical Sciences, Tehran, Iran; 2 Chronic Respiratory Diseases Research Center, National Research Institute of Tuberculosis and Lung Diseases (NRITLD), Shahid Beheshti University of Medical Sciences, Tehran, Iran; 3 Department of Cardiology, Clinical Research Development Unit Farshchian Heart Center, School of Medicine, Hamadan University Medical Sciences, Hamadan, Iran; 4 Department of Epidemiology, School of Public Health, Hamadan University of Medical Sciences, Hamadan, Iran; 5 Pharmacology Toxicology Department, Pharmacy School, Hamadan University of Medical Sciences, Hamadan, Iran; 6 Internist School of Medicine, Hamedan University of Medical Sciences, Hamadan, Iran

**Keywords:** Cardiac Autonomic Neuropathy, Cardiovascular Autonomic Symptoms, Diabetic Peripheral Neuropathy, Gabapentin, Heart Rate Variability

## Abstract

**Background:**

Cardiac autonomic neuropathy (CAN) is one of the most significant complications of diabetes mellitus (DM) and is characterized by reduced heart rate variability (HRV). The CAN frequently coexists with peripheral neuropathy.

**Objectives:**

We attempted to determine whether gabapentin has any impact on HRV in individuals suffering from painful diabetic peripheral neuropathy (PDPN).

**Methods:**

In this double-blinded randomized controlled trial, 30 patients with painful peripheral neuropathy were enrolled. Fifteen patients in the intervention group were randomized to receive gabapentin capsules and capsaicin placebo cream, while 15 patients in the control group were randomized to receive gabapentin placebo capsules and capsaicin cream. The diagnosis of PDPN was established using the Neuropathy Symptom Scale (NSS), neuropathy disability score (NDS), and Visual Analogue Scale (VAS). The HRV was evaluated with the standard deviation of normal-normal beats (SDNN) via 24-hour Holter monitoring of heart rate.

**Results:**

Of the 30 randomized patients, 26 (86.7%) were included in the analysis (n = 15 intervention, n = 11 control). There were no statistically significant differences in age, sex, or Body Mass Index (BMI) between the two groups. Gabapentin increased the average level of SDNN by 11 ms in the intervention group and decreased by 6 ms in the control group. The between-group standardized mean difference (MD, Hedges’ g) was 0.756 (95% CI = -0.04 - 1.5, P-value = 0.069), indicating a moderate effect that was marginally significant.

**Conclusions:**

Our findings in this small pilot trial suggest that gabapentin therapy may improve cardiovascular autonomic function and increase HRV in patients suffering from diabetic painful peripheral neuropathy. However, more studies with larger populations are needed to ultimately prove this.

## 1. Background

Diabetic neuropathy (DN) is a chronic complication of diabetes mellitus (DM), affecting up to 50% of individuals with type 1 or type 2 DM (T1 DM or T2 DM) (1). The DN can manifest in three primary forms: Peripheral, autonomic, and painful neuropathy (2). The most frequent type of autonomic neuropathy, identified as cardiac autonomic neuropathy (CAN), is characterized by damage to the autonomic nerve fibers that innervate the heart and blood vessels (3). The prevalence of CAN in type 1 and type 2 diabetes varies, ranging from 17% to 73%, depending on clinical and demographic variables (4). The five-year mortality rates of CAN for individuals with type 1 and type 2 diabetes range from 16 - 50% (5).

The risk of silent myocardial ischemia and mortality in patients with DM is increased up to twofold, which is related to reduced cardiovascular autonomic function, as evaluated by heart rate variability (HRV) (6). Therefore, HRV is a useful index of CAN because it reflects the variability in the time between consecutive heartbeats (7). The standard deviation of normal-normal beats (SDNN) is considered the gold standard for assessing cardiac risk. It is recorded over a 24-hour period and reflects the overall performance of both the sympathetic autonomic nervous system (SANS) and the parasympathetic autonomic nervous system (PANS) (8, 9). The SDNN also provides data on the overall HRV. When the HRV is characterized by large and irregular fluctuations, the SDNN values are relatively high (9).

Unfortunately, a standardized treatment protocol for CAN is currently lacking. Diagnosis at an early stage, behavioral modifications, treatment of dyslipidemia (DLP), tighter glycemic control, antioxidants, especially α-lipoic acid (ALA), vitamins, management of cardiovascular risk factors, and in extreme cases, treatment of orthostatic hypertension are all part of the treatment options (10). The administration of thromboxane prostacyclin analogs, A2 blockers, and phosphodiesterase-5 (PDE5) inhibitors, along with the combined prescription of ALA, ω-3 polyunsaturated fatty acids (ω-3 PUFAs), and dihomo-γ-linolenic acid (DGLA), are novel prospective strategies for the treatment of CAN (10).

In 2000, gabapentin, initially developed as an antiepileptic medication, was also approved in the United Kingdom for the treatment of neuropathic pain (11). To our knowledge, only limited research has examined whether gabapentin affects autonomic DN, despite its routine use for diabetic peripheral neuropathy (DPN) and its well-known anticonvulsant and analgesic properties.

## 2. Objectives

Since DPN and diminished HRV often occur together (12), patients with painful diabetic peripheral neuropathy (PDPN) were enrolled in our study, aiming to evaluate the impact of gabapentin on HRV.

## 3. Methods

### 3.1. Study Design and Patient Population

**Figure 1.**
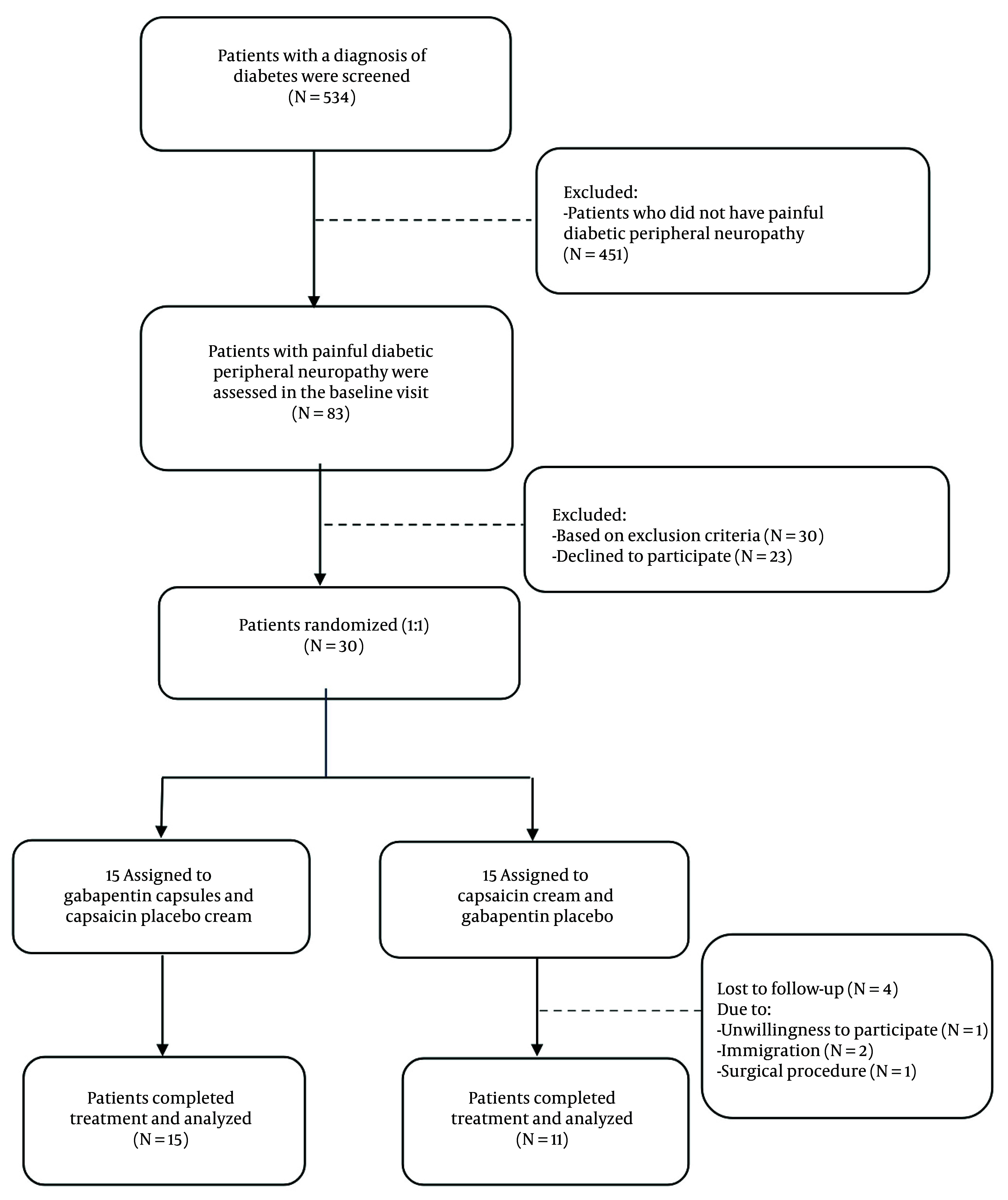
Patient inclusion flow chart

This is a double-blind, randomized controlled trial that evaluated the effect of gabapentin on HRV. Between July 2020 and May 2021, 534 diabetic patients were referred to Shahid Beheshti Hospital (Hamadan, Iran). Thirty patients with DM suffering from painful peripheral neuropathy were consecutively included in the study according to the inclusion criteria (Figure 1). The study protocol was approved by the local Ethics Committee (ethics code: D/P/3044/9/35/16), and the participants provided written informed consent.

The primary sample size was calculated as 24 patients, based on the one study (13). The power is assumed to be 80%, and the type 1 error is considered 5%. Some patients (20%) were predicted to miss follow-up sessions for 3 months, calculated as 15 people. Accordingly, 26 patients who fulfilled the criteria and completed the follow-up sessions were evaluated out of the 30 initially enrolled.

### 3.2. Inclusion and Exclusion Criteria

The diagnosis of DPN was established using the neuropathy disability score (NDS) and the Neuropathy Symptom Scale (NSS). Electromyography/nerve conduction velocity (EMG/NCV) testing was not feasible at our center; moreover, our goal was to emphasize clinical findings. Patients were considered to have DPN if NDS ≥ 6, or if NSS ≥ 5 with NDS 3 - 5. Using the Visual Analogue Scale (VAS), patients with a pain score ≥ 4 for ≥ 2 hours per day were classified as having painful DPN. Given that nerve conduction studies (NCS) are regarded as the reference standard for confirming DPN but are resource-intensive and not ideal for routine screening, validated clinical scores were used in this trial (13).

The NSS comprises 17 yes/no items (8 motor weakness, 5 sensory, 4 autonomic; score range 0 - 17, higher = worse) (13). The NDS quantifies neuropathy severity on a 0 - 10 examination-based scale (higher = worse) derived from ankle reflexes and large-fiber sensation (vibration with tuning fork, pin-prick, and temperature) (14). Both scores have been validated against NCS; reported diagnostic accuracy versus NCS is approximately 61 - 82% sensitivity and 67 - 75% specificity for NSS, and 61 - 92% sensitivity and 48 - 100% specificity for NDS, varying with cut-offs and case-mix (15, 16).

Patients with a history of cardiac arrhythmias, valvular heart disease, heart block, symptomatic ischemic heart disease, open-heart surgery, major depression, end-stage renal disease, hypersensitivity to gabapentin, hypersensitivity to capsaicin, not responding to capsaicin, allergy to pepper, and those with a history of taking antiarrhythmic drugs, beta-blockers, tricyclic antidepressants, phenothiazines, herbal remedies, and complementary medicines, pregnancy and lactation, or having an unstable general condition were excluded. The CAN was evaluated using standard Ewing's tests and heart rate variation during physical examination (at rest, tachycardia, and orthostatic hypotension). Our previous article covered the specifics of the research technique (17, 18).

### 3.3. Randomization and Blinding

Eligible patients were assigned to either the intervention or control groups in a 1:1 ratio via a block randomization method with permuted blocks of 4 and allocation sequence concealment. The intervention group received gabapentin capsules and capsaicin placebo cream. On the other hand, the control group was supplied with capsaicin cream to reduce pain and gabapentin placebo capsules. Considering that it is ethically impossible to give a placebo to a patient who is in pain, we preferred to use topical capsaicin to improve the patient's pain because it was readily available and well tolerated, providing minimal systemic absorption with no negative functional or neurological effects (19).

Gabapentin therapy was initiated as a single 100 mg dose on day 1, 200 mg/day on day 2, and 300 mg/day on day 3. The dose was subsequently titrated up as needed for neuropathic symptom relief to a total daily dose of 1800 mg. Capsaicin cream was also used 3 times a day. The participants were randomly assigned to receive one of two identically labeled packs (A or B) in a double-blinded design. Pack A contained capsaicin cream plus placebo gabapentin; pack B contained gabapentin plus placebo capsaicin cream. The placebos were matched to their active counterparts in appearance and packaging to preserve blinding. The pharmacologist was responsible for drug categorization. Participants, treating clinicians, caregivers, and outcome assessors were unaware of the pack contents throughout the study.

### 3.4. Clinical Endpoints

All patients were referred to a cardiac electrophysiologist for 24-hour Holter monitoring using the Schiller MT-101 Holter recorder (Suprima Holter System, DMS, USA) to assess SDNN as the primary endpoint of this study. The secondary endpoints were Body Mass Index (BMI), blood pressure, and pulse rate in a standing position (after standing for 2 minutes) and in a supine position (after 15 minutes of rest). The patients were followed monthly by telephone or in-person for the proper use of drugs and possible side effects. Those who discontinued taking the medication for more than 30% of the trial period or doses were excluded. The assigned treatment was planned to be continued until a 90-day follow-up, and at the end of the trial, all endpoints were reevaluated.

### 3.5. Statistical Analysis

Categorical data are reported by frequency and percentage, whereas continuous variables are reported as means and standard deviations (SDs). A *t*-test was applied to estimate the mean difference (MD) of average SDNN, blood pressure, heart rate, and BMI between intervention and control groups. Additionally, Hedges' g through the psychometrica website was utilized to estimate the standard mean difference (SMD) of the outcomes with a 95% confidence interval (CI). According to Jacob Cohen's work in his book, the interpretation areas of Hedges' g are defined as small for values between 0.2 and 0.5, medium for values between 0.5 and 0.8, and large for values greater than 0.8 (20). Additionally, comparisons were made via the chi-square test for categorical variables. P-values less than 5% were considered statistically significant. Statistical analysis was performed using SPSS 26.

## 4. Results

Among the 534 diabetic patients referred to the endocrine clinic from July 2020 to May 2021, 15% (83 patients) had PDPN, and 30 patients were randomly assigned to intervention and control groups (Figure 1). A total of 26 patients, including 15 in the intervention group and 11 in the control group, completed this study. Differences in age, sex, BMI, heart rate, and systolic and diastolic blood pressure between the two groups before the intervention were not statistically significant (Table 1). 

**Table 1.** Comparisons of Age, Sex, Average Body Mass Index, Heart Rate, and Blood Pressure in the Study Population Before the Intervention ^a^

Variables	Interventions	Controls	P-Value
**Age (y)**	59 ± 10.11	52.55 ± 7.6	0.088
**Female; No. (%)**	13 (86.7)	10 (90.9)	0.738
**BMI (kg/m** ^**2**^ **)**	27.02 ± 4.83	26.32 ± 5.22	0.830
**Standing SBP ** ^**b**^ ** (mmHg)**	134.67 ± 19.77	140.00 ± 19.10	0.497
**Supine SBP (mmHg)**	139.67 ± 23.02	145.00 ± 22.80	0.563
**Standing DBP ** ^**c**^ ** (mmHg)**	79.67 ± 8.95	80.91 ± 10.44	0.747
**Supine DBP (mmHg)**	81.00 ± 9.67	81.82 ± 12.50	0.852
**Supine HR ** ^**d**^ ** (beat/min)**	86.47 ± 13.45	88.82 ± 8.15	0.613
**Standing HR (beat/min)**	94.33 ± 14.45	94.64 ± 9.97	0.953

Abbreviation: BMI, Body Mass Index.

^a^ Values are expressed as mean ± standard deviation (SD) unless indicated.

^b^ Systolic blood pressure.

^c^ Diastolic blood pressure.

^d^ Heart rate.

**Figure 2.**
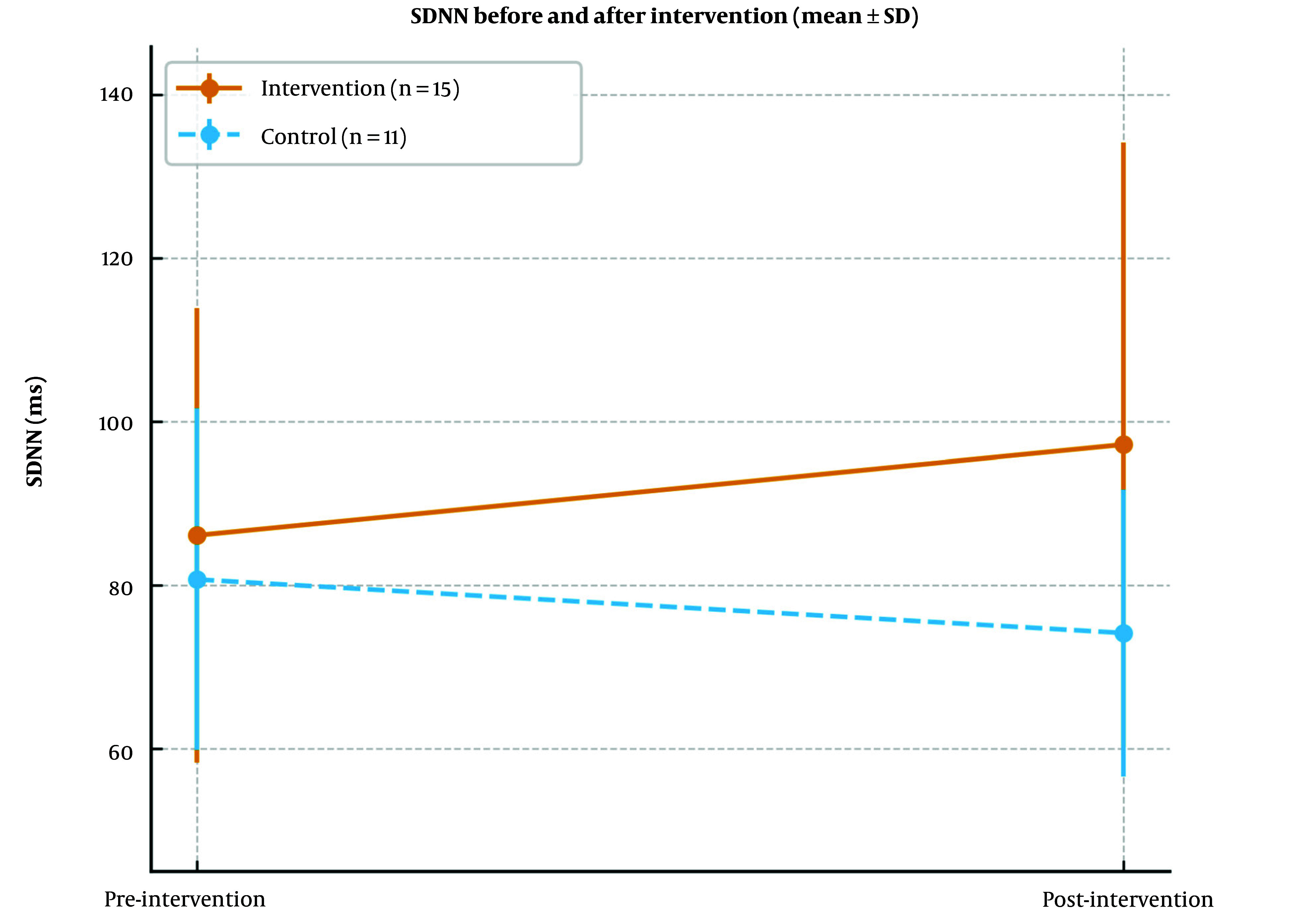
Standard deviation of normal-normal beats (SDNN, ms) before and after the intervention in each group [mean ± standard deviation (SD), error bars indicate SDs; intervention: n = 15; control: n = 11].

Baseline SDNN was 86.13 ± 27.78 ms in the intervention group versus 80.73 ± 20.88 ms in the control group (P = 0.593). After 12 weeks, SDNN was 97.20 ± 36.97 ms versus 74.18 ± 17.58 ms. Gabapentin increased the average level of SDNN by 11 ms in the intervention group and decreased by 6 ms in the control group. The between-group SMD was calculated via Hedges' g, which was 0.756 (95% CI = -0.04 to 1.56; P = 0.069), indicating a moderate effect that was marginally significant (20, 21) (Figure 2). 

The probability of improving the SDNN to more than 100 levels in the intervention group is 27% greater than that in the control group [RR = 0.73, 95% CI (0.39 to 1.38), P = 0.658; Table 2]. 

**Table 2.** Standard Deviation of Normal-Normal Beats Categories After Intervention (ms) ^a^

Categorires	Intervention (N = 15)	Control (N = 11)	P-Value
**< 100 ms**	10 (66.7)	9 (81.8)	0.658
**≥ 100 ms**	5 (33.3)	2 (18.2)	

^a^ Values are expressed as No. (%).

The SMDs of the final standing systolic and diastolic blood pressures, as well as the final supine diastolic blood pressure, between the intervention and control groups, were calculated via Hedges' g. The values obtained were 0.456 (95% CI = -0.331 to 1.244, P-value = 0.262), 0.439 (95% CI = -0.348 to 1.226, P-value = 0.279), and 0.337 (95% CI = -0.446 to 1.121, P-value = 0.403), respectively. Based on conventional benchmarks for Hedges' g, gabapentin had a small effect (20) (Table 3). 

**Table 3.** Comparison of the Average Body Mass Index, Heart Rate, and Blood Pressure of the Study Population After the Intervention

Variables	Intervention Mean	Control Mean	MD	SMD	P-Value
**BMI**	76.93	72.22	-4.70	0.43	0.290
**Standing SBP ** ^**a**^	136.20	145.91	9.70	0.456	0.262
**Supine SBP **	136	140.00	4.00	0.186	0.643
**Standing DBP ** ^**b**^	68.00	76.36	8.36	0.439	0.279
**Supine DBP**	78.67	80.00	1.33	0.337	0.403
**Standing HR ** ^**c**^	83.73	85.09	1.35	0.143	0.723
**Supine HR**	78.07	79.45	1.38	0.186	0.643

Abbreviations: MD, mean difference; SMD, standard mean difference; BMI, Body Mass Index.

^a^ Systolic blood pressure.

^b^ Diastolic blood pressure.

^c^ Heart rate.

Six patients in the intervention group and three patients in the control group experienced further complications (Table 4). 

**Table 4.** Adverse Events ^a^

Complications	Intervention (N = 15)	Control (N = 11)	Time to Onset	Outcome
**Any complication (≥ 1)**	6 (40.0)	3 (27.3)		
**Dyspepsia**	2 (13.3)	0 (0.0)	1th day	Resolved
**Blisters**	1 (6.7)	0 (0.0)	7th day	Resolved
**Vertigo**	1 (6.7)	0 (0.0)	1th day	Resolved
**Flatulence**	1 (6.7)	0 (0.0)	5th day	Resolved
**Dysesthesia (right buttock)**	1 (6.7)	0 (0.0)	3th day	Resolved
**Hypersomnia**	0 (0.0)	1 (9.1)	3th day	Resolved
**Contact dermatitis**	0 (0.0)	1 (9.1)	1th day	Resolved
**Burning reaction to capsaicin cream**	0 (0.0)	1 (9.1)	1th day	Resolved

^a^ Values are expressed as No. (%).

## 5. Discussion

In this study, we evaluated the effect of gabapentin on HRV, which is currently an acceptable index of cardiac autonomic dysfunction. This drug is widely used in PDPN, so we also investigated its ability to improve CAN. Our study demonstrated improvement in cardiac autonomic function in patients with diabetic painful peripheral neuropathy who took gabapentin for three months. The CAN is one of the most underappreciated yet significant consequences of DM (3). Patients with CAN often show no symptoms (22) or may experience nonspecific symptoms such as dizziness, palpitations, and light-headedness (23). Syncope, myocardial infarction, and orthostatic intolerance are all examples of potentially life-threatening complications that may arise (22, 24).

Gabapentin is a structural analogue of GABA; however, it does not bind GABA receptors and does not affect GABA synthesis or reuptake. It crosses the blood–brain barrier and can influence neurotransmission. Its principal pharmacologic action is high-affinity binding to sites associated with presynaptic voltage-gated calcium channels, particularly the α2δ-1 subunit (25). At presynaptic terminals, α2δ-1 binding reduces presynaptic Ca^2+^ entry and suppresses the release of excitatory neurotransmitters (e.g., glutamate) from primary afferents, consistent with the analgesic profile of gabapentinoids (25, 26). There are four genes (α2δ-1 through α2δ-4) that encode α2δ subunits, which are expressed in various tissues. In cardiac muscle, the α2δ-1 isoform predominates. Furthermore, α2δ-1 is highly expressed in skeletal muscle and brain (27). Gabapentinoids interact with the auxiliary α2δ-1 and α2δ-2 subunits of calcium channels; however, only α2δ-1 has been associated with the development of neuropathy in animal studies. Transgenic models have identified α2δ-1 as a crucial mediator of the analgesic properties of gabapentinoids.

By attenuating nociceptive afferent drive into central autonomic circuits (e.g., brainstem nuclei such as the nucleus tractus solitarius), gabapentin may lower sympathetic arousal and favor vagal modulation, which could manifest as higher time-domain HRV (e.g., SDNN) in painful neuropathy (28, 29). Accordingly, gabapentinoids are considered important first-line treatments for several neuropathic pain conditions of central and peripheral origin (30). In patients with painful DPN, daily doses of gabapentin between 1,800 mg and 3,600 mg may reduce pain and improve sleep disturbances (31).

Prior evidence on HRV is limited but suggestive. In a 2008 study by Necip Ermis et al., some HRV parameters differed after three months of gabapentin therapy in diabetic patients with peripheral neuropathy compared with baseline (e.g., SDNN increased from 106.2 ± 29.8 to 119.4 ± 25.0 ms; P = 0.016) (12). In addition, after seven months of gabapentin treatment for Rett syndrome, physicians observed an improvement in SDNN from baseline in an 18-year-old patient (although HRV was not the primary endpoint, this may indicate an effect on SDNN) (32). Conversely, a study by Pan et al. reported that long-term use of gabapentin or pregabalin in patients with DN was associated with increased risks of myocardial infarction, heart failure, stroke, peripheral vascular disease, pulmonary embolism, and deep-vein thrombosis (33).

In our trial, treatment was comparatively short (12 weeks), and no cardiovascular adverse events were observed, suggesting that such risks may be less likely with short-term exposure. Therefore, when prescribing gabapentin or pregabalin for long-term use in DN, clinicians should weigh patient tolerability and analgesic efficacy against the potential for increased cardiovascular risk (33).

Because there is currently no established disease-modifying therapy for CAN, we examined whether gabapentin could improve CAN, given its common use in DPN. This pilot had a relatively small sample size and a short follow-up period (12 weeks), which limited statistical power, generalizability, and the ability to assess the durability of HRV changes or long-term cardiovascular safety signals. The HRV analysis was restricted to the time domain (SDNN) exported by the Holter platform; frequency-domain metrics such as low frequency/high frequency (LF/HF) and direct sympathetic measures were not obtained because specialized equipment was unavailable at our center. Neuropathy classification relied on NSS/NDS rather than EMG/NCV due to limited electrophysiology availability, which may miss subclinical disease and introduce misclassification relative to nerve-conduction confirmation. Accordingly, these findings should be regarded as preliminary.

Confirmation in larger, multicenter trials is warranted, ideally with cellular–molecular investigations and comprehensive autonomic testing. Future studies may also compare patients with and without chronic diabetic complications. Despite these limitations, few studies have evaluated the effect of gabapentin on HRV, and this trial contributes to the limited evidence base.

### 5.1. Conclusions

Our findings in this small pilot trial suggest that gabapentin therapy in patients with PDPN may improve cardiac autonomic function. However, further research with larger populations should be conducted to substantiate this claim.

## Data Availability

The datasets used and/or analyzed during the current study are available from the corresponding author upon reasonable request.

## Clinical Trial Registration Code:

IRCT201308179014N18.

## References

[1] Cernea S, Raz I (2021). Management of diabetic neuropathy.. Metabolism..

[2] Didangelos T, Karlafti E, Kotzakioulafi E, Margariti E, Giannoulaki P, Batanis G (2021). Vitamin B12 Supplementation in Diabetic Neuropathy: A 1-Year, Randomized, Double-Blind, Placebo-Controlled Trial.. Nutrients..

[3] Vinik AI, Ziegler D (2007). Diabetic cardiovascular autonomic neuropathy.. Circulation..

[4] Williams S, Raheim SA, Khan MI, Rubab U, Kanagala P, Zhao SS (2022). Cardiac Autonomic Neuropathy in Type 1 and 2 Diabetes: Epidemiology, Pathophysiology, and Management.. Clin Ther..

[5] Eleftheriadou A, Williams S, Nevitt S, Brown E, Roylance R, Wilding JPH (2021). The prevalence of cardiac autonomic neuropathy in prediabetes: a systematic review.. Diabetologia..

[6] Gupta R, Maheshwari A, Verma N, Narasingan SN, Tripathi K (2023). InSH Consensus Guideline for the Management of Hypertension, 2023.. Hypertension Journal..

[7] Martinez PF, Okoshi MP (2018). Heart Rate Variability in Coexisting Diabetes and Hypertension.. Arq Bras Cardiol..

[8] Duque A, Mediano MFF, De Lorenzo A, Rodrigues LJ (2021). Cardiovascular autonomic neuropathy in diabetes: Pathophysiology, clinical assessment and implications.. World J Diabetes..

[9] Hinde K, White G, Armstrong N (2021). Wearable Devices Suitable for Monitoring Twenty Four Hour Heart Rate Variability in Military Populations.. Sensors (Basel)..

[10] Serhiyenko VA, Serhiyenko AA (2018). Cardiac autonomic neuropathy: Risk factors, diagnosis and treatment.. World J Diabetes..

[11] Bennett MI, Simpson KH (2004). Gabapentin in the treatment of neuropathic pain.. Palliat Med..

[12] Ermis N, Gullu H, Caliskan M, Unsal A, Kulaksizoglu M, Muderrisoglu H (2010). Gabapentin therapy improves heart rate variability in diabetic patients with peripheral neuropathy.. J Diabetes Complications..

[13] Carmichael J, Fadavi H, Ishibashi F, Shore AC, Tavakoli M (2021). Advances in Screening, Early Diagnosis and Accurate Staging of Diabetic Neuropathy.. Front Endocrinol (Lausanne)..

[14] Tavakoli M, Boulton AJ, Efron N, Malik RA (2011). Increased Langerhan cell density and corneal nerve damage in diabetic patients: role of immune mechanisms in human diabetic neuropathy.. Cont Lens Anterior Eye..

[15] Asad A, Hameed MA, Khan UA, Ahmed N, Butt MU (2010). Reliability of the neurological scores for assessment of sensorimotor neuropathy in type 2 diabetics.. J Pak Med Assoc..

[16] Kamel SR, Hamdy M, Abo Omar HA, Kamal A, Ali LH, Abd Elkarim AH (2015). Clinical diagnosis of distal diabetic polyneuropathy using neurological examination scores: correlation with nerve conduction studies.. Egyptian Rheumatology and Rehabilitation..

[17] Vasheghani M, Sarvghadi F, Beyranvand MR (2019). The association between cardiac autonomic neuropathy and diabetes control.. Diabetes Metab Syndr Obes..

[18] Vasheghani M, Sarvghadi F, Beyranvand MR, Emami H (2020). The relationship between QT interval indices with cardiac autonomic neuropathy in diabetic patients: a case control study.. Diabetol Metab Syndr..

[19] Vinik AI, Perrot S, Vinik EJ, Pazdera L, Jacobs H, Stoker M (2016). Capsaicin 8% patch repeat treatment plus standard of care (SOC) versus SOC alone in painful diabetic peripheral neuropathy: a randomised, 52-week, open-label, safety study.. BMC Neurol..

[20] Gallardo-Gomez D, Richardson R, Dwan K (2024). Standardized mean differences in meta-analysis: A tutorial.. Cochrane Evid Synth Methods..

[21] Olsson-Collentine A, van Assen M, Hartgerink CHJ (2019). The Prevalence of Marginally Significant Results in Psychology Over Time.. Psychol Sci..

[22] Peters E, Itani M, Kristensen AG, Terkelsen AJ, Kroigard T, Tankisi H (2023). Cardiovascular autonomic neuropathy in patients with type 2 diabetes with and without sensorimotor polyneuropathy.. J Peripher Nerv Syst..

[23] Wadhera S, Rastogi A, Dutta P, Gupta A, Bhadada SK (2022). Age and Disease Duration Independent Cardiac Autonomic Neuropathy in Patients with Diabetic Foot Complications: Case-Control Study.. Indian J Endocrinol Metab..

[24] Agashe S, Petak S (2018). Cardiac Autonomic Neuropathy in Diabetes Mellitus.. Methodist Debakey Cardiovasc J..

[25] Yasaei R, Katta S, Patel P (2025). Gabapentin..

[26] Dolphin AC (2018). Voltage-gated calcium channel alpha (2)delta subunits: an assessment of proposed novel roles.. F1000Res..

[27] Tuluc P, Kern G, Obermair GJ, Flucher BE (2007). Computer modeling of siRNA knockdown effects indicates an essential role of the Ca2+ channel alpha2delta-1 subunit in cardiac excitation-contraction coupling.. Proc Natl Acad Sci U S A..

[28] Forte G, Troisi G, Pazzaglia M, Pascalis V, Casagrande M (2022). Heart Rate Variability and Pain: A Systematic Review.. Brain Sci..

[29] Dampney RA, Horiuchi J, Tagawa T, Fontes MA, Potts PD, Polson JW (2003). Medullary and supramedullary mechanisms regulating sympathetic vasomotor tone.. Acta Physiol Scand..

[30] Patel R, Dickenson AH (2016). Mechanisms of the gabapentinoids and alpha 2 delta-1 calcium channel subunit in neuropathic pain.. Pharmacol Res Perspect..

[31] Cohen K, Shinkazh N, Frank J, Israel I, Fellner C (2015). Pharmacological treatment of diabetic peripheral neuropathy.. P T..

[32] Gualniera L, Singh J, Fiori F, Santosh P (2021). Emotional Behavioural and Autonomic Dysregulation (EBAD) in Rett Syndrome - EDA and HRV monitoring using wearable sensor technology.. J Psychiatr Res..

[33] Pan Y, Davis PB, Kaebler DC, Blankfield RP, Xu R (2022). Cardiovascular risk of gabapentin and pregabalin in patients with diabetic neuropathy.. Cardiovasc Diabetol..

